# Molecular Mechanisms of the Endocannabinoid System with a Focus on Reproductive Physiology and the Cannabinoid Impact on Fertility

**DOI:** 10.3390/ijms26157095

**Published:** 2025-07-23

**Authors:** Patrycja Kalak, Piotr Kupczyk, Antoni Szumny, Tomasz Gębarowski, Marcin Jasiak, Artur Niedźwiedź, Wojciech Niżański, Michał Dzięcioł

**Affiliations:** 1 Department of Reproduction and Farm Animals, Wroclaw University of Environmental and Life Sciences, 50-375 Wroclaw, Poland; patrycja.kalak@upwr.edu.pl (P.K.); wojciech.nizanski@upwr.edu.pl (W.N.); 2 Department of General and Experimental Pathology, Wroclaw Medical University, 50-368 Wroclaw, Poland; piotr.kupczyk@umw.edu.pl; 3 Department of Food Chemistry and Biocatalysis, Faculty of Biotechnology and Food Science, Wroclaw University of Environmental and Life Sciences, 50-375 Wroclaw, Poland; antoni.szumny@upwr.edu.pl; 4 Department of Animal Physiology and Biostructure, Faculty of Veterinary Medicine, Wroclaw University of Environmental and Life Sciences, 50-375 Wroclaw, Poland; tomasz.gebarowski@upwr.edu.pl; 5 Department of Internal Diseases with Clinic for Horses, Dogs, and Cats, Wroclaw University of Environmental and Life Sciences, pl. Grunwaldzki 47, 50-366 Wroclaw, Poland; marcin.jasiak@upwr.edu.pl (M.J.); artur.niedzwiedz@upwr.edu.pl (A.N.)

**Keywords:** endocannabinoid system, cannabinoid receptors, cannabinoids, reproduction, breast cancer

## Abstract

The endocannabinoid system (ECS) is a complex neuromodulatory network involved in maintaining physiological balance through interactions with various neurotransmitter and hormonal pathways. Its key components—cannabinoid receptors (CBRs)—are activated by endogenous ligands and exogenous cannabinoids such as those found in the *Cannabis sativa* plant. Although cannabinoids like cannabidiol (CBD) have garnered interest for their potential therapeutic effects, evidence regarding their safety, particularly for reproductive health, remains limited. This review summarizes the structure and molecular mechanisms of the ECS, its role in reproductive physiology—including its interactions with the hypothalamic–pituitary–gonadal axis (HPG axis), gametogenesis, implantation, and lactation—and the possible consequences of cannabinoid exposure for fertility. In addition, we focus on the involvement of the ECS and cannabinoids in breast cancer, highlighting emerging evidence on their dual role in tumor progression and therapy. These insights emphasize the need for further research to better define the therapeutic potential and risks associated with cannabinoid use in reproductive health and breast cancer.

## 1. Introduction

Hemp (*Cannabis sativa* L.) is one of the oldest cultivated plants, used for various industrial (e.g., fiber production), nutritional (e.g., seed oil production), and medicinal purposes. Archaeological and historical records indicate that cannabis has been employed in traditional medicine across many ancient cultures. In China, hemp seeds are consumed for their nutritional value and used medicinally to treat malaria, rheumatism, constipation, and other ailments [1,2]. In India, cannabis was recognized as a sacred plant and applied in treating pain, seizures, and inflammation [2,3]. Middle Eastern and African societies used cannabis to manage infections, gastrointestinal disorders, and complications during childbirth [4,5]. The routes of administration and methods of preparation varied depending on the region and purpose of use, including oral ingestion, smoking, and topical application.

In the 19th century, cannabis attracted attention in Western medicine. Notably, William B. O’Shaughnessy introduced cannabis into European pharmacology as a treatment for tetanus, cholera, rabies, and rheumatic conditions [3,6]. Around the same time, French psychiatrist Jacques-Joseph Moreau explored its psychoactive effects in mental illness and neurological disorders [7]. However, inconsistent effects and a lack of standardized dosing, along with increasing social and regulatory concerns, led to a decline in medical use. Cannabis was removed from the U.S. Pharmacopeia in 1941 and later classified as a Schedule I controlled substance, significantly hindering research for several decades [3].

The discovery and isolation of Δ9-tetrahydrocannabinol (THC) and cannabidiol (CBD) in the 1960s by Raphael Mechoulam and colleagues [8,9] marked the beginning of modern cannabinoid research. These breakthroughs paved the way for the identification of the endocannabinoid system (ECS)—a conserved lipid signaling network consisting of cannabinoid receptors (CBRs), endogenous ligands such as anandamide (AEA) and 2-arachidonoylglycerol (2-AG), and enzymes involved in their synthesis and degradation [10,11]. The ECS is now known to play a crucial role in maintaining homeostasis by regulating processes within the nervous, endocrine, immune, and reproductive systems [12,13,14,15,16,17].

With growing interest in the therapeutic potential of cannabinoids—especially CBD—numerous products are marketed as natural alternatives to conventional pharmaceuticals. However, scientific evidence regarding their safety and long-term effects remains limited, particularly in relation to reproductive health. Most available data concern THC, while studies on the safety profile of CBD and other plant-derived cannabinoids (phytocannabinoids) are still emerging [11,13]. Additionally, cannabinoids have been investigated for their involvement in tumor biology, with particular emphasis on breast cancer, where they appear to exert both pro- and anti-tumor effects depending on the context [18,19,20,21,22].

The aim of this review is to provide a comprehensive overview of the molecular architecture and signaling mechanisms of the ECS, its physiological role in reproduction, and the effects of exogenous cannabinoids on fertility. Special attention is given to the role of ECS in breast cancer, with a focus on its potential as a therapeutic target and the associated biological risks.

## 2. The Endocannabinoid System

ECS plays a crucial role in regulating and maintaining homeostasis across virtually all physiological processes in the body [12,13,14,15,16,17]. It achieves this balance primarily through the production of endogenous ligands (known as endocannabinoids or endogenous cannabinoids), which act to restore physiological equilibrium when homeostasis is disrupted. Central to ECS function are two principal G protein-coupled receptors (GPCRs): cannabinoid receptor type 1 (CB1) and cannabinoid receptor type 2 (CB2). Due to their distinct tissue distribution and involvement in diverse biological pathways, these receptors make them promising targets for therapeutic intervention [23].

Although the ECS was discovered relatively recently, it is now recognized as a highly conserved signaling system across nearly all species [10]. Beyond CBRs and their endogenous ligands, the ECS encompasses enzymes responsible for the synthesis, transport, and degradation of endocannabinoids. Key enzymes include fatty acid amide hydrolase (FAAH) and monoacylglycerol lipase (MAGL), which inactivate endocannabinoids, thereby tightly regulating ECS activity [11].

### 2.1. Cannabinoid Receptors

#### 2.1.1. CB1 and CB2: Types, Structure, and Mechanisms of Action

CB1 and CB2 are integral components of the ECS and belong to class A (rhodopsin-like) GPCRs [24,25]. Both receptors primarily couple with Gi/o proteins and mediate their effects by inhibiting adenylate cyclase activity, leading to decreased intracellular cyclic adenosine monophosphate (cAMP) levels [26,27,28]. CB1 has also been shown to couple with Gq and, in some contexts, Gs proteins, although the latter remains less defined for CB2 [29,30].

CB1 is predominantly expressed in the central nervous system, (CNS), while CB2 is more abundant in immune-related tissues. However, both receptors are also present in peripheral organs, including reproductive tissues such as the ovary, uterus, testis, and placenta, where they modulate local endocrine, immune, and cellular functions [14,26,29,30].

Upon activation by endocannabinoids or exogenous cannabinoids, both receptors initiate intracellular signaling cascades, including the mitogen-activated protein kinase (MAPK) pathway and the phosphatidylinositol-3-kinase (PI3K)/AKT (Protein Kinase B) pathway. These signaling routes regulate key cellular processes such as proliferation, differentiation, apoptosis, migration, and inflammation [18,31,32,33,34,35]. In reproductive tissues, these pathways are involved in folliculogenesis, spermatogenesis, implantation, and placental development, while in cancer, they are implicated in tumor growth, angiogenesis, and immune evasion [19,20,21,22,27,36,37].

Although CB1 and CB2 possess complex structural features—including seven transmembrane helices and distinct ligand binding domains—this review focuses on their signaling relevance in reproductive and oncological contexts. Notably, ligand binding modulates calcium and potassium channel activity, influencing neurotransmitter and hormone release, which may affect gonadotropin regulation and reproductive homeostasis [38,39,40,41,42,43].

Emerging data also suggest a functional interplay between the ECS and other signaling systems, such as the dopaminergic and oxytocinergic pathways, which are critical for neuroendocrine regulation of reproduction and sexual behavior [44,45,46,47].

#### 2.1.2. Location of CB1 and CB2

**Figure 1:**
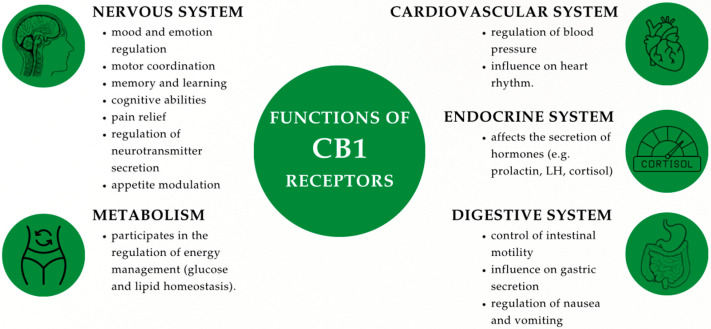
Functions of CB1 receptors.

CBRs have been characterized so far in various vertebrate species. Although present throughout virtually all tissues, a certain localization tendency of individual CBRs was observed. Despite some interspecies variation, CB1 shows a conserved expression pattern, with its highest density consistently found in the CNS, where it plays a key role in regulating neuronal activity, and to a lesser extent in the periphery (e.g., adrenal glands, bone marrow, heart, lungs, prostate) [11,25,48,49,50,51]. Studies have shown that these receptors are most highly expressed in axons and presynaptic terminals of neurons in the amygdala, hippocampus, cortex, basal ganglia outflow tracts, and cerebellum [52], which is consistent with previous observations indicating that cannabinoids have a marked effect on locomotor activity, cognitive functions, and memory performance, as well as pain, appetite, and emotions [34,53]. Upon activation, the CB1 receptor increases the activity of calcium and potassium ion channels, which consequently modulates the release of neurotransmitters. Functionally significant expression of this receptor has also been detected in cardiovascular, hormonal, and digestive tissues—it has been noted, among other things, that it reduces intestinal motility and secretion, supports anti-inflammatory effects (by inhibiting the release of pro-inflammatory cytokines), helps control vomiting, and regulates cell growth and survival, influencing proliferation and apoptosis, which is important in the case of cancer and neurodegenerative diseases. This wide distribution of CB1 receptors in the body underlies their diverse functions [10,34,43,53,54,55,56,57,58,59,60,61,62,63,64,65,66,67] (Figure 1).

**Figure 2:**
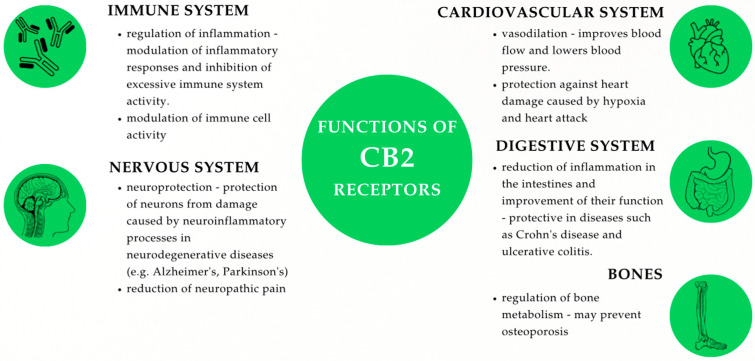
Functions of CB2 receptors.

In contrast, research indicates that CB2 receptors are predominantly expressed in the cells of the digestive and immune systems. This distribution partly accounts for the immunomodulatory properties of cannabinoids [23,25,58,68,69,70,71,72]. Moreover, CB2 expression was also detected in the CNS, more precisely in astrocytes and microglia [73]. The mechanism of their action involves the inhibition of adenylyl cyclase activity via Gi/Goα subunits and interaction with stimulatory Gαi/o proteins, leading to altered intracellular cAMP levels and modulation of downstream MAPK signaling cascades, which significantly affect both mature and developing tissues [74,75,76,77]. Signaling through these receptors plays a major role in modulating inflammation. It is also associated with neuroprotective effects and the maintenance of bone mass [10,13,78,79]. Additionally, research has already been carried out on the effects of CB2 agonists in slowing down neurodegenerative diseases (e.g., Alzheimer’s disease) [80] (Figure 2).

Furthermore, although data remain limited, preliminary studies in humans and various animal species demonstrate the presence and functional importance of both receptors in reproductive tissues (i.e., ovaries, corpus luteum (CL), uterus, placenta, testes, vas deferens) and mammary glands [10,65,81,82,83,84,85,86,87,88,89,90,91,92,93,94,95,96,97,98,99,100,101,102,103,104] (Table 1). A detailed discussion of this topic is provided in Section 3 (The Role of the Endocannabinoid System and Cannabinoids in Reproductive Processes) and Section 4 (Meaning of the endo- and exocannabinoids for the mammary tumor (development and treatment)) in this article.

### 2.2. Cannabinoid Ligands

Cannabinoid ligands are molecules that interact with CRBs modulating their activity and initiating downstream cellular signaling. These ligands can be broadly classified into three major groups: (1) endogenous cannabinoids, which are synthesized naturally within the body and include compounds such as anandamide and 2-AG; (2) plant-derived cannabinoids, primarily found in *Cannabis sativa*, including THC, CBD, and other minor phytocannabinoids; and (3) synthetic cannabinoids, (SCs)**,** structurally diverse compounds developed to mimic or modulate activity of CBRs. Each group exhibits distinct affinity, efficacy, and receptor selectivity profiles, influencing a wide range of physiological and pathological processes.

The term exogenous cannabinoids is sometimes used functionally to refer to both phytocannabinoids and SCs, as they originate outside the organism.The following subsections explore each group in detail, including their biosynthesis and receptor interactions.

#### 2.2.1. Endogenous Cannabinoids (Endocannabinoids)

The identification of CB1 and CB2 receptors enabled the discovery of natural endocannabinoids—molecules that are neurotransmitters responsible for activating receptors. Most attention has been paid to research on AEA and 2-AG [105,106,107,108]. These molecules are released postsynaptically and exert a homeostatic function by activating CBRs, resulting in the rapid inhibitory modulation of neurotransmitters responsible for various biological processes such as pain, inflammation, immunity, bone growth, and anxiety [109]. Endocannabinoids bind to receptors, inhibit voltage-gated calcium channels and adenylate cyclase activity, and stimulate MAPK pathways [105,110,111]. To achieve this, the production and inactivation/hydrolysis of endocannabinoids is tightly controlled by the precise action of metabolic enzymes: FAAH (AEA degradation) and MAGL (2-AG degradation) [10,79]. Additionally, studies have shown that endocannabinoids bind to the CB1 with slightly greater affinity than to the CB2 receptor [72]. Similarly, inhibition of cAMP by AEA is greater for the CB1 [112], whereas inhibition of cAMP production by 2-AG is greater for CB2 [113]. Interestingly, analyses have shown that 2-AG occurs in the brain in concentrations 170 times higher than AEA, and additionally, it acts as a full agonist, causing the characteristic effects associated with cannabinoid agonists [114]. In addition to CBRs, both AEA and 2-AG also interact with other receptors [115,116,117,118].

Beyond their biochemical and homeostatic roles, endocannabinoids such as AEA and 2-AG have also been implicated in the regulation of reproductive functions, including follicular development, sperm capacitation, embryo implantation, and uterine contractility, highlighting their physiological relevance beyond the endocannabinoid signaling in the nervous system (see Section 3).

#### 2.2.2. Plant-Derived Cannabinoids (Phytocannabinoids)

**Figure 3:**
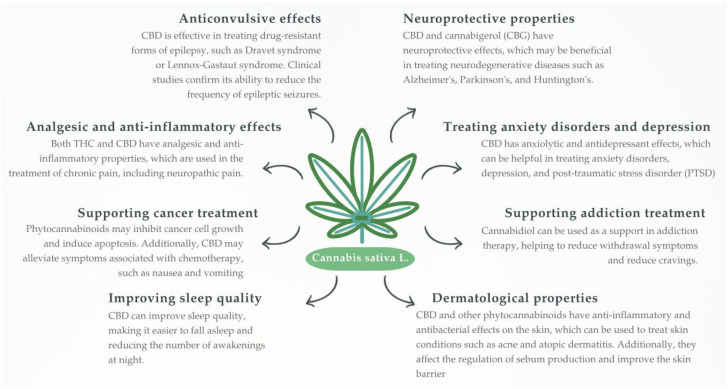
The drawing schematically shows *Cannabis sativa* L. plant and its known or potential therapeutic properties of phytocannabinoids derived from this plant. It should be remembered that the main source of phytocannabinoids is the flowers.

In recent years, growing societal acceptance, widespread use of cannabis (both recreational and medical), and legalization in some countries have prompted scientists to examine its constituents and their effects on the body [119]. *Cannabis sativa* L. is an Asian plant with a rich chemical composition; in addition to primary metabolites (e.g., fatty acids and amino acids), it also contains secondary metabolites, including phytocannabinoids—plant-derived exogenous cannabinoids capable of interacting with CBRs. Among these classes, cannabinoids are well-studied compounds whose structure resembles that of endocannabinoids produced by the human body and effectively mimics their action. Phytocannabinoids exhibit a wide range of therapeutic properties, including analgesic, muscle relaxant, immunosuppressive, anti-inflammatory, antiallergic, sedative, mood-elevating, appetite-stimulating, antiemetic, and anticancer effects [11,120,121,122,123,124,125,126] (Figure 3).

Recent analyses indicate that exogenous cannabinoids can directly influence tumor growth by inducing cancer cell death and/or inhibiting proliferation [127] and indirectly reduce the expression of vascular endothelial growth factor (VEGF) [128]. At physiological concentrations, they may also support the recovery of damaged macromolecules and organelles, thereby helping maintain the structural integrity and functional balance of lysosomes [129,130]. These effects result from activation of CBRs or competition between endocannabinoids and metabolic enzymes [131]. Experiments have identified important aspects of this mechanism and provided insight into how cannabinoid agonists induce biased signaling [131]. It is also important to note that upon binding extracellular ligands such as phytocannabinoids, GPCRs interact with specific subsets of heterotrimeric G proteins, which in turn activate or inhibit various effector enzymes and ion channels. Among phytocannabinoids, two compounds have been studied in detail: THC and CBD [132].

##### Δ9-Tetrahydrocannabinol

Initially, the rapid increase in cannabis use worldwide (e.g., as an analgesic, anticonvulsant, sedative, or antiemetic for nausea in pregnant women), combined with its psychoactive effects and strong affinity for CB1 receptors in the CNS, made THC a primary subject of scientific research. It acts as a partial agonist of CB1, and its effects include downregulation of the secondary messenger cAMP via inhibition of adenylate cyclase [58,133]. Among other properties, THC can inhibit the production of prostaglandin E2 (PGE2) [134]. As noted, it also has strong narcotic properties mediated by central CBRs, which are well documented. The observed psychotropic effects include euphoria, relaxation, and antinociception [49]. Because endocannabinoids are crucial for proper brain development, these effects may result from cannabinoids disrupting endocannabinoid homeostasis, despite structural differences between these groups of compounds [135,136]. Current studies show that adolescent cannabinoid use may lead to structural, functional, and histological brain alterations that could underlie some long-term behavioral and psychological harms [137]. It has also been shown that cannabinoid use in this group significantly slows the learning process [138]. As a partial rather than full CB1 agonist, THC exhibits lower cytotoxicity compared to SCs (e.g., CP-55,940) and therefore has a better safety profile [133,139].

##### Cannabidiol

In recent years, interest in CBD has grown significantly due to its psychoactive but non-intoxicating properties, along with considerable clinical potential as an alternative to conventional therapies for various health disorders in humans and animals, including its FDA (Food and Drug Administration)-approved use in the treatment of epilepsy [13]. It is the second most abundant phytocannabinoid in cannabis, accounting for up to 40% of the dry weight of some varieties [140]. Like THC, CBD has neuroprotective, antioxidant, and antimicrobial effects [126,141]. Although structurally similar [142], these two compounds have different mechanisms of action. While THC is a partial agonist of CB1 and CB2 with direct interaction, CBD has lower affinity for both receptors and is a CB1 antagonist. Moreover, Laprairie et al. (2015) reported that CBD largely acts as a negative allosteric modulator (NAM) of CB1 receptor agonism by THC and 2-AG [143]. CBD’s minimal agonism at CBRs may account for its negligible psychotropic effect [144]. In addition, cannabidiol has the ability to interact with other receptors, e.g., opioids, which has been tested on rodents and in vitro [144]. Additionally, unlike THC, CBD can activate the receptor transient receptor potential vanilloid 1 (TRPV1), primarily found in the nervous system and involved in the generation and transmission of pain signals, as well as inhibit FAAH activity and AEA reuptake [145,146]. Preliminary clinical studies also indicate that CBD may alleviate symptoms of anxiety, motor and cognitive disorders, pain, and epileptic seizures. However, the specific mechanisms of action of its biological effects remain unclear [78].

Cannabis likely contains smaller compounds beyond the two main cannabinoids discussed. As they are believed to have greater pharmacological activity, scientific research on these compounds is expected to grow significantly in the coming years. Interestingly, one important parameter influencing the biological activity of THC-like cannabinoids is the length of the C3 alkyl chain. A minimum of three carbon atoms is required for receptor binding. Activity increases with chain length up to eight carbons, after which it begins to decline. When the number of carbon atoms is greater than eight, the activity begins to decrease again [147].

##### Other Minor Phytocannabinoids

Beyond the major phytocannabinoids such as THC and CBD, *Cannabis sativa* produces over 100 minor cannabinoids, including cannabigerol (CBG), cannabichromene (CBC), cannabinol (CBN), and tetrahydrocannabivarin (THCV) [10,72,73]. These compounds have been reported to exert anti-inflammatory, antioxidant, and neuroprotective effects [10,72,77].

However, their biological activity appears to be lower and less well characterized than that of THC or CBD. To date, little is known about their influence on reproductive processes or hormone-related cancers. Available data are mostly preclinical and focus on general physiological responses rather than specific reproductive or oncological endpoints [72,78]. 

#### 2.2.3. Synthetic Cannabinoids

Research on CBRs structure, supported by bioinformatic and cheminformatic models, enabled the synthesis of a chemically diverse group of compounds known as SCs [148]. Following the identification and cloning of CBRs, numerous structurally diverse substances were studied for their ability to bind to and activate these receptors, leading to large-scale synthesis. These compounds contributed to studies on both classical and non-classical receptors within the ECS, as well as to the development of potential therapies for diseases such as multiple sclerosis and HIV (Human Immunodeficiency Virus) [149]. Unfortunately, some of these substances were misused as narcotics and appeared on the black market [150].

On the international pharmaceutical market, SC-based drugs that are structurally and functionally similar to THC are available. Dronabinol, approved by the FDA in 1985, is used to treat anorexia associated with HIV/AIDS (Acquired Immunodeficiency Syndrome) and chemotherapy-induced nausea. Studies are ongoing into its use for obstructive sleep apnea syndrome (OSAS) and chronic pain in palliative care. However, it can cause side effects such as neuropsychiatric and hemodynamic disorders, seizures, addiction, and paradoxical nausea and vomiting [151]. In Canada and the United Kingdom, nabilone is approved for use. It causes milder side effects, such as drowsiness, dizziness, dry mouth, and fatigue, and is used for treating chronic pain, insomnia, spasticity, and symptoms of neurodegenerative diseases like Alzheimer’s and Parkinson’s [152,153,154].

In addition, a SC, JT11, has recently been characterized as a highly selective CB2 receptor agonist. It shows promising anti-inflammatory properties via modulation of signaling pathways and cytokine release in immune cells, such as peripheral blood mononuclear cells. Unlike many classical cannabinoids that primarily act on CB1 receptors, the CB2 selectivity of JT11 reduces psychoactive effects while potentially offering therapeutic benefits in inflammatory conditions. Its ability to induce proapoptotic activity and regulate immune responses highlights JT11 as a novel candidate for further investigation into cannabinoid-based therapies targeting CB2 receptors [155].

Recreational SCs, misleadingly marketed as synthetic marijuana or fake weed, are sold under names such as “Spice” (Europe), “K2” (USA), and “Bonzai” or “Jamaica” (Turkey) as supposedly safe alternatives to marijuana. In reality, they often contain high-affinity CB1 agonists, resulting in more intense, violent, and dangerous effects than natural cannabis products. Consequently, they are classified as new psychoactive substances (NPSs) [156,157,158].

The synthetic THC analog HU-210, considered a classical cannabinoid, has a narcotic potency at least 100 times greater than THC. Another group of SCs, labeled as “non-classical”, includes cyclohexylphenols or 3-arylcyclohexanols (CP compounds), developed as potential pain relievers in the 1980s. Other SC variants unrelated to THC have also emerged, such as aminoalkylindoles like naphthoylindoles (e.g., JWH-018), phenylacetylindoles (e.g., JWH-250), and benzoylindoles (e.g., AM-2233). JWH-018 is likely the most well-known SC, with a potency three times that of THC [106,159].

Recent SCs exhibit increasing structural diversity, likely designed to bypass legal restrictions on earlier substances. While the trade of cannabis and THC-containing products is strictly regulated under anti-drug laws, controlling SCs is far more challenging, raising their potential for misuse [160].

### 2.3. Non-Classical Cannabinoid Receptors

In addition to CB1 and CB2, several other receptors—commonly referred to as non-classical CBRs—have been proposed to participate in cannabinoid signaling. These include GPRs such as GPR55 and GPR18, ion channels like TRPV1, and nuclear receptors including peroxisome proliferator-activated receptors (PPARs) [10,85,87,90]. These receptors have been implicated in diverse physiological processes, including inflammation, nociception, energy metabolism, and immune responses [10,85,88].

Although their expression has been observed in some reproductive tissues [10,89,90], current knowledge of their roles in reproductive physiology or hormone-dependent cancers, such as breast cancer, is limited and largely derived from in vitro studies or animal models. Further research is necessary to clarify their significance in these contexts.

## 3. The Role of the Endocannabinoid System and Cannabinoids in Reproductive Processes

The relationship between cannabinoids and reproductive function can be considered in the context of endogenous interactions between components of the ECS and the hormonal pathways that regulate reproductive processes. It should also be examined in relation to the effects of exogenous cannabinoids, which, by binding to CBRs expressed at various levels of the reproductive regulatory axis, may significantly modulate these processes. The ECS has been shown to play a critical role in regulating both male and female reproductive functions, including gametogenesis, fertility, fertilization, implantation, embryonic and fetal development, gonadal maturation, libido, and lactation (Table 2 and Table 3). Disruption of ECS activity may lead to reproductive and mammary gland dysfunction. Despite the growing interest in this field, studies investigating the influence of exogenous cannabinoids on reproduction—and thus their safety—remain limited. Existing research has primarily focused on the effects of THC in humans and in rodent models. Although some controversies have emerged, most available studies suggest a potentially adverse impact of these compounds on reproductive outcomes.

### 3.1. ECS and Cannabinoids in Female Reproductive Processes

#### 3.1.1. ECS and Its Physiological Connections with the Hypothalamic–Pituitary–Gonadal Axis(HPG Axis): Influence on the Secretion of Sex Hormones and Menstrual Cycle Regulation

ECS is present throughout the HPG axis and modulates key reproductive processes in both sexes. ECS components, especially the CB1 receptor, are found in GnRH (Gonadotropin-Releasing Hormone)-secreting neurons of the hypothalamus, where endocannabinoids like AEA inhibit GnRH release either directly or indirectly via GABAergic interneurons [161]. This leads to decreased secretion of downstream hormones, including luteinizing hormone (LH), follicle-stimulating hormone (FSH), and prolactin (PRL) [161,162,163,164].

Disruption or absence of CBRs impairs hormonal release and reproductive function [162]. In females, ECS dysregulation has been linked to menstrual irregularities, anovulation, and delayed ovulation, associated with altered receptor expression in the hypothalamus, anterior pituitary, ovaries, and endometrium [163,164]. Chronic exposure to THC has been shown to reduce GnRH, LH, FSH, and PRL levels [165,166,167,168,169,170,171]. In animal models (rats, rhesus monkeys), THC exposure delays puberty onset, disrupts estrous cycles, and affects endometrial stromal differentiation [111,172,173], possibly via altered ovarian and pituitary signaling [174].

Bidirectional regulation between sex hormones and ECS components has also been demonstrated. Estradiol (E2) modulates ECS elements in the uterus, and AEA levels fluctuate with the menstrual cycle [84,173,175,176]. Importantly, reduced FAAH activity—responsible for AEA degradation—has been associated with miscarriage [111,177]. ECS dysregulation, especially due to exogenous cannabinoids, may contribute to reproductive pathologies such as polycystic ovary syndrome (PCOS) [178] and is also linked to altered CB1/CB2 receptor expression in endometrial disorders [179,180].

#### 3.1.2. ECS in Female Reproductive Tissues and Gametes

CB1 and CB2 receptors are expressed in ovarian follicles at multiple stages—primordial, primary, and secondary—suggesting their role in folliculogenesis and oocyte maturation [161]. FAAH is localized in the cytoplasm of oocytes and granulosa cells across these stages, and luteal cells are immunopositive for both CB1 and FAAH [181,182], indicating ECS involvement in follicular selection, ovulation, and corpus luteum (CL) function.

Pirone et al. (2017) identified stage-specific localization of ECS components in feline reproductive tissues [181]. CB1 was found exclusively in the granulosa cells of tertiary follicles, while FAAH was broadly expressed from early follicular stages and in various ovarian compartments. In the fallopian tube, FAAH was present in both ciliated and non-ciliated epithelial cells, whereas CB1 was restricted to ciliated cells—possibly serving as a species-specific cellular marker [181,182].

Although in vivo studies have not consistently demonstrated ECS-mediated inhibition of progesterone (P4) secretion by the CL [111,182,183,184,185,186,187,188,189], in vitro bovine studies suggest that CBR activation may antagonize luteotrophic signals (LH, Prostaglandin E1 (PGE1), PGE2). This effect is mediated by the inhibition of adenylate cyclase, activation of Protein Kinase C (PKC), and increased intracellular calcium levels, all of which promote luteolysis [190,191,192,193,194,195,196]. ECS involvement in luteal regression has also been demonstrated in sheep [79].

#### 3.1.3. ECS in Fertilization, Pregnancy, and Lactation

ECS plays a critical role during fertilization and early pregnancy. Disruption of AEA synthesis in the ovary or uterus impairs embryo development and implantation [197]. In vitro studies show that high AEA levels inhibit blastocyst development and attachment [198] and that FAAH is expressed in preimplantation embryos [111]. Cannabinoids may delay or block embryo transport through the oviduct, potentially increasing the risk of ectopic pregnancy [101,199,200].

All ECS components are present in the uterus. AEA inhibits oxytocin-induced contractions, promoting uterine quiescence during pregnancy. As parturition approaches, AEA is converted to prostaglandins via arachidonic acid, promoting labor onset [161]. CB2, FAAH, AEA, and TRPV1 have been detected in the placenta [96,161,201]. Notably, CB1 expression is higher in placentas from cesarean deliveries compared to spontaneous labor, suggesting a role in parturition pathways [161]. AEA is active during early pregnancy, aiding implantation, while 2-AG levels—much higher than AEA—peak during brain development [161], underscoring the importance of the ECS in fetal neurodevelopment.

Maternal cannabis consumption has been associated with increased risks of preterm birth, low birth weight, neonatal intensive care admission, and neurodevelopmental disorders [180,186,187,188,189,202]. These effects likely result from ECS pathway disruptions critical to implantation, placental function, and fetal development. Nonetheless, some human studies have shown no significant fertility decline among cannabis users [203,204], underscoring the need for further research.

Finally, the ECS influences lactation through the regulation of PRL secretion. Chronic THC exposure suppresses PRL and impairs milk production [133,199]. Both CB1 and CB2 are expressed in mammary glands, and THC metabolites readily cross the placenta and appear in breast milk, potentially affecting neonatal development [196,203,205,206].

These findings highlight the need for tightly regulated ECS activity during the peri-implantation period and throughout pregnancy and lactation.

### 3.2. ECS and Cannabinoids in Male Reproductive Processes

#### 3.2.1. ECS in Male Reproductive Tissues and Male Gametes

Components of the ECS exhibit distinct localization patterns in spermatozoa. The CB1 receptor is distributed along the sperm head membrane, midpiece, and tail, where it contributes to membrane stability. In contrast, the CB2 receptor is predominantly localized in the sperm head membrane [161]. The ECS plays a crucial role in regulating key sperm functions such as capacitation and the acrosome reaction. Schuel et al. [99] demonstrated that appropriate levels of AEA in seminal plasma maintain sperm in a metabolically quiescent state, while its gradual decline within the female reproductive tract facilitates sperm activation.

The ECS is fundamentally involved in male reproductive physiology. CB1 and CB2 receptors as well as AEA have been identified in human testicular tissue, including Sertoli and Leydig cells, and in spermatozoa, where receptor localization differs [207,208,209]. In sperm, activation of the CB1 receptor has been shown to reduce both viability and motility [98,210,211]. Additionally, TRPV1 signaling is present in Sertoli cells, where its activation induces apoptosis [212].

Based on these findings, an inverse correlation between seminal endocannabinoid levels and sperm motility has been established [213]. Therefore, disturbances in the endocannabinoid balance in seminal plasma can adversely affect spermatogenesis and male fertility.

#### 3.2.2. ECS in Spermatogenesis and Male Reproductive Health

Experimental studies indicate that cannabinoids exert detrimental effects on the male reproductive system. THC penetrates the blood–testis barrier in a concentration-dependent manner that correlates with serum levels. Similarly to its effects in females, it disrupts the HPG axis, which regulates sperm production and sex hormone secretion [214]. Chronic cannabinoid exposure has been associated with decreased LH levels, while FSH levels remain largely unchanged and testosterone (T) concentrations show inconsistent alterations [215,216].

The negative influence of cannabis on semen quality is well-documented in both humans and rodent models and includes reductions in sperm count and concentration, as well as alterations in morphology and motility [216,217,218]. Furthermore, studies focused on CBD alone have demonstrated its gonadotoxic effects on mammalian spermatogenesis via multiple mechanisms. Long-term cannabinoid use has also been linked to clinical manifestations including gynecomastia, erectile dysfunction, and reduced potency [219].

### 3.3. ECS and Cannabinoids in Gonadal Development and Steroidogenesis

CB1 and CB2 are expressed in the gonads, where they modulate cellular differentiation, proliferation, and steroidogenesis. Exogenous cannabinoid exposure during fetal or perinatal periods may cause long-term endocrine disruptions, including altered testicular architecture and testosterone production [220].

### 3.4. The Role of the ECS in Libido Regulation

ECS plays a pivotal role in modulating sexual motivation and behavior via central and peripheral mechanisms. The key neurochemical pathways involved include the dopaminergic and oxytocinergic systems, which serve as essential mediators of sexual drive and reward processing. Activation of the ECS influences these pathways, thereby affecting libido and sexual performance.

THC, the primary psychoactive cannabinoid, exhibits biphasic effects on sexual motivation: at lower doses, THC may enhance libido by stimulating dopaminergic signaling, whereas higher doses or chronic use tend to suppress sexual desire and function. This dose-dependent modulation highlights the complex influence of cannabinoids on sexual behavior [220,221,222,223]. However, available data remain limited, particularly with regard to species-specific responses, highlighting the need for further investigation to elucidate the precise mechanisms and translational relevance.

### 3.5. Sex-Specific Differences in Reproductive ECS Activity

Sex-based differences in ECS activity are evident in central reproductive centers. In rodents, CB1 density is higher in the male hypothalamus and pituitary, while females exhibit elevated circulating AEA levels [161]. Moreover, the menstrual cycle influences ECS activity, as receptor and enzyme expression levels fluctuate, contributing to sex-specific physiology and ECS-related reproductive disorders.

### 3.6. ECS Receptors and Endocrine-Disrupting Chemicals (EDCs)

ECS is present in both female and male gonads. Disruptions of this system can lead to dysfunctions in the reproductive organs and gametes. This can occur when external substances bind to ECS receptors, leading to unfavorable modulation. This may result from interactions with EDCs, such as phthalates. It is now known that these substances can interact with components of the ECS. It has been shown, for example, that DINP (diisononyl phthalate) impairs the ECS gene expression profile in both male and female gonads of zebrafish. In humans, germ cell tumors, which are a component of testicular dysgenesis syndrome (TDS), are associated with the malignant transformation of precursor cells into germ cell neoplasia in situ (GCNIS). According to Barchi et al. [224], cannabinoid signaling may play a crucial role in this process. Another substance that can interfere with the ECS is bisphenol A (BPA), which binds to CB1.

### 3.7. Cannabinoids and Non-Steroidial Anti-Inflammatory Drugs (NSAIDs) in the Context of Reproduction

It is worth noting that cannabinoids, like NSAIDs, have been shown to act as selective cyclooxygenase type 2 (COX-2) inhibitors [225]. In mammals, prostaglandins are synthesized from arachidonic acid through the action of cyclooxygenase (COX), specifically its two isoforms—cyclooxygenase type 1 (COX-1) and COX-2—which are differentially expressed [226,227]. COX-1 activity has been observed in virtually all mammalian tissues, and the prostaglandin produced by this isoform plays essential roles in normal physiological processes [226]. In contrast, COX-2 expression is induced by factors such as cytokines, mitogens, and endotoxins, with its primary function being the increased production of prostaglandins during inflammatory states [227].

Prostaglandins are also crucial bioregulators of reproductive functions. Numerous studies suggest that, from a biophysical and biochemical perspective, the process of ovulation resembles a typical inflammatory response [228,229,230,231]. The pre-ovulatory surge of gonadotropins enhances the expression of COX, primarily COX-2, in granulosa and theca cells of ovarian follicles. In many species, increased COX-2 expression during the pre-ovulatory period leads to elevated prostaglandin levels (mainly prostaglandin E (PGE) within the follicle). This, in turn, raises P4 concentrations, highlighting the significance of these components in ovulation and their supportive role in the early stages of CL development [229,232,233,234]. Furthermore, it has already been demonstrated that COX-2 expression is critical for oocyte maturation and early embryonic development, while prostaglandins play a vital role in preparing the endometrium for embryo implantation [235,236,237,238,239].

Numerous studies conducted on various species indicate that COX-2 inhibitors can have both positive and negative effects on fertility. Research on the use of selected NSAIDs during the periovulatory period, conducted in humans, primates, rats, mice, rabbits, pigs, sheep, and cows, predominantly suggests a significantly negative impact of these drugs on fertility [240,241,242]. In humans, primates, and rabbits, NSAIDs were found to disrupt ovulation, leading some researchers to propose their use as an alternative to hormonal contraception [240,241,242]. In women, cases of “luteinized unruptured follicle syndrome” were reported, which resolved after discontinuation of the drugs [243]. In dogs, NSAIDs reduced P4 levels but did not negatively affect fertility [244]. Conversely, other studies on the administration of COX-2 inhibitors during various phases of the ovarian cycle showed no negative impact on reproductive capacity in animals [245,246,247] and even improved outcomes in embryo transfer procedures in cattle and mares [245,247,248]. However, their harmful effects were observed in early pregnancy in Holstein heifers [249]. There are also suggestions that the influence of NSAIDs depends on their anti-inflammatory properties—some drugs more effectively inhibited ovulation, while others had no significant impact [250].

Given the role of cyclooxygenase in the ovarian cycle, the similar mechanisms of action of cannabinoids and NSAIDs, and the known impact of NSAIDs on reproductive processes, cannabinoid ligands may also have potential significance in this context. Preliminary scientific analyses have already begun exploring this hypothesis. Notably, the findings of Cosentino et al. (2022), who investigated the effects of CBD on the expression and function of COX-1 and COX-2 in human neutrophils, revealed that CBD profoundly inhibits COX-1 and COX-2 mRNA (messenger Ribonucleic Acid) expression without significantly affecting PGE2 production [251]. However, these aspects require further, more detailed research.

## 4. Meaning of the Endo- and Exocannabinoids for the Mammary Tumor (Development and Treatment)

### 4.1. The Role of the ECS in Cancer Biology

As already mentioned, endocannabinoids present in vertebrates and invertebrates can modulate the activity of proteins involved in cell proliferation, differentiation, and apoptosis. For this reason, it has been hypothesized that the ECS may be involved in controlling cancer cell survival [252]. Given the widespread processes of carcinogenesis (including in the breast) and the mechanisms of the ECS, scientists have been investigating the therapeutic potential of cannabinoids, which has been widely described in the literature. However, the results of research on the role of the ECS in the development of cancer are contradictory. Some data suggest that the ECS may be excessively activated, which may promote tumor growth [253]. Conversely, other studies indicate that the ECS has anticancer effects by inhibiting tumor growth [254,255]. There is already a theory that this effect may be dose-dependent—low doses of cannabinoids promote cell proliferation, while higher doses exhibit antiproliferative effects [256].

### 4.2. Interaction Between ECS and Hormonal Regulation in Breast Cancer

It is already known that the ECS, together with sex hormones, plays an important role in the pathogenesis of breast cancer, modulating key processes such as proliferation, angiogenesis, metastasis, and response to treatment [18,19]. Previous studies have shown that, as in other cancers, the ECS undergoes significant changes in breast cancer. These changes are closely related to tumor aggressiveness. Elevated levels of endocannabinoids, as well as altered expression of CBRs and enzymes responsible for endocannabinoid metabolism, have been associated with more aggressive cancer phenotypes, emphasizing the involvement of the ECS in its progression [20].

The CB2 receptor is clearly overexpressed in breast cancer, especially in HER2 (Human Epidermal Growth Factor Receptor 2 Positive) tumors, where it occurs in about 90% of cases. Increased CB2 expression correlates with poorer prognosis and increased tumor aggressiveness. CB2 mRNA levels have been found to be higher in estrogen receptor negative (ER−)/progesterone receptor negative (PR−) tumors compared with estrogen receptor positive (ER+)/progesterone receptor positive (PR+) tumors and are also elevated in high-grade histologies [21,22,256]. Such differential expression of CBRs in tumor subtypes could provide a molecular basis for personalized cannabinoid-based therapies. However, CB2 expression in ER+ tumors is associated with better prognosis. In addition, increased N-acylphosphatidylethanolamine, the precursor of AEA, as well as higher levels of MAGL have been observed, particularly in ductal carcinomas [18,257,258].

### 4.3. Cannabinoids in Triple-Negative Breast Cancer (TNBC)

Most studies of cannabinoids in breast cancer have focused TNBC. Studies have shown that cannabinoid sensitivity in human breast cancer cell cultures correlates with tumor aggressiveness; ER− cell lines are more sensitive than ER+ lines [256]. CBD, one of the most studied phytocannabinoids, has been shown to reduce proliferation in MDA-MB-231 cells, potentially through activation of TRPV1 and other unknown targets [19,259]. Furthermore, CBD induces apoptosis in human breast cancer MDA-MB-231 cells by engaging CB1, CB2, and TRPV1. This apoptosis is a result of endoplasmic reticulum stress, inhibition of the AKT/mTOR (Mechanistic Target of Rapamycin) pathway, and activation of autophagy [260]. The ability of CBD to inhibit cell survival has also been linked to interactions with pathways involving PPARs, mTOR, and cyclin D1, as demonstrated by Sultan et al. 2018 [261]. In turn, THC has been reported to promote proliferation in MDA-MB-231 cells [262]. CBD also exhibits antimigratory and anti-invasive properties in various TNBC cell lines, mediated by inhibition of EGF (Epidermal Growth Factor)/EGFR (Epidermal Growth Factor Receptor) signaling and downstream cascades involved in cell proliferation, survival, and inflammatory responses [263]. Given the involvement of these pathways in tumor progression, ECS-targeted modulation may offer dual benefits—attenuating tumor growth while minimizing adverse systemic effects. Studies in mouse xenograft models have confirmed the tumor-inhibiting effects of CBD, although CBD resistance has been observed in some cases [259,264]. CBD also reduces metastatic potential by downregulating inhibitor of differentiation protein 1 (Id-1) via modulation of the ERK (Extracellular Signal-Regulated Kinase) pathway. Sustained activation of ERK is associated with growth inhibition, while transient activation promotes cell proliferation [264,265]. In addition, CBD increases the production of reactive oxygen species (ROS), which may further inhibit Id-1 activity, increasing apoptosis [259,260,264,265]. However, TNBC cell survival has also been shown to be dependent on ROS levels, suggesting a complex role of ROS in the breast cancer microenvironment [266]. These findings suggest that targeting oxidative stress pathways through ECS modulation could represent a novel therapeutic avenue, particularly in cancers with high ROS-related aggressiveness, such as TNBC. SCs such as JWH-133 have shown significant antitumor effects in TNBC models. JWH-133 reduced tumor growth and angiogenesis in both xenograft and genetically modified mouse models [267]. These compounds inhibit cell migration through mechanisms involving CB1 and CB2. They interfere with key regulators of cytoskeletal remodeling and adhesion, thereby reducing the ability of cells to migrate and invade surrounding tissues [268,269]. In addition, they inhibit the COX-2/PGE2 axis and modulate the ERK signaling pathway [267,270]. By influencing both immune and vascular components of the tumor microenvironment, ECS modulation may reshape the tumor milieu toward an anti-cancer state. SCs such as Met-F-AEA, WIN 55,212-2, and JWH-133 have also been shown to induce cell cycle arrest and reduce metastatic potential, suggesting broad antiproliferative and antimetastatic properties [267,269]. While mechanistic details continue to emerge, the clinical relevance of ECS modulation in oncology is underscored by these preclinical findings.

### 4.4. Cannabinoids in HER2+ Breast Cancer

In HER2+ breast cancer, overexpression of CB2 has been associated with poorer patient outcomes [22]. Preclinical studies suggest that CB2 activation in HER2+ tumor models may mediate antitumor effects, making CB2-targeted therapies promising for inhibiting tumor growth [256]. The most prominent studies in this context have used MMTV-neu (Mouse Mammary Tumor Virus-neu) mice, a well-established model for HER2+ tumors. This study demonstrated that THC reduced tumor growth, angiogenesis, and metastasis by inducing apoptosis and inhibiting the AKT signaling pathway. Similar effects were observed with JWH-133, a CB2-selective agonist [271].

### 4.5. Cannabinoids in Luminal A (ER+) Breast Cancer

Luminal A tumors, predominantly ER+, are the most commonly diagnosed subtype of breast cancer. Cannabinoid studies in this subtype have yielded promising results. AEA has been shown to inhibit basal, PRL-induced, and nerve growth factor (NGF)-induced proliferation in MCF-7 and EFM-19 cells via activation of CB1. This inhibition involved cell cycle arrest and activation of the Raf-1/ERK/MAPK pathway, resulting in decreased expression of PRL and the NGF receptor [256,272]. Similar antiproliferative effects were observed when THC acted on EVSA-T (Estrogen-Responsive Mammary Tumor Cell Line) cells via activation of CB2, mediated by inhibition of CDK1 (Cyclin-Dependent Kinase 1) and activation of JunD (component of the AP-1 transcription factor) [273,274]. However, there is conflicting evidence, with some studies showing that THC may promote cell proliferation in MCF-7 cells through mechanisms involving Act1 (NF-κB Activating Protein 1) and HER2 (Human Epidermal Growth Factor Receptor 2) [262,275,276,277]. On the other hand, CBD has been shown to reduce cell survival and induce apoptosis in T-47D cells via modulation of PPAR, mTOR, and cyclin D1 pathways [262]. In addition, CBD can induce endoplasmic reticulum stress in MCF-7 cells, leading to cell death via increased ROS and calcium levels, likely mediated by TRPV1 activation [278]. As for other cannabinoids, CBG has been reported to reduce proliferation in MCF-7 cells [259], while CBN appears to stimulate proliferation in the same model [276,277]. Furthermore, THC analogues have been shown to bind to ER\estrogen receptor alpha (*ERα*), potentially activating this receptor, which has been implicated in antitumor activity. THC has also been reported to prevent E2-induced proliferation in MCF-7 cells via an ER-independent mechanism [279]. A recent study by Almada et al. showed that AEA, CBD, and THC reduced cell viability and disrupted cell cycle progression in MCF-7aro cells. AEA and THC induced apoptosis, while CBD promoted autophagy-mediated cell death. These cannabinoids also inhibited aromatase activity, further supporting their potential role in modulating estrogen signaling [280,281,282]. Interestingly, THC has been shown to increase *estrogen receptor beta* (ERβ) expression, which is considered beneficial in the treatment of ER+ breast cancer [283].

### 4.6. Cannabinoids in Combination with Hormonal Therapies

A promising area of research is the study of combining cannabinoids with endocrine therapies. Tamoxifen, a selective estrogen receptor modulator (SERM), has been identified as a modulator of CB1 and CB2, acting as an inverse agonist of these receptors. Studies have shown additive antiproliferative effects when tamoxifen is combined with THC or cannabinoid-based formulations in T-47D cells [284,285]. However, conflicting data suggest that THC may promote MCF-7 cell growth in combination with aromatase inhibitors (AIs) [275]. Overall, cannabinoids have shown potential to inhibit tumor growth and prevent metastasis in ER+ breast cancer. These effects are primarily achieved through cell cycle arrest, apoptosis, and autophagy, driven by activation of CB1 and CB2 and modulation of pathways such as mTOR and Raf-1/ERK/MAPK.

Therefore, cannabinoids, both natural and synthetic, represent a promising group of therapeutic compounds whose potential requires further clinical investigation.

## 5. Summary

As the stigma surrounding cannabis use continues to decline and legal access becomes more widespread, understanding the molecular mechanisms of CBRs and their ligands is increasingly important. This is particularly true as clinical and preclinical reports on phytocannabinoid activity accumulate. Although the ECS remains under investigation, numerous scientific studies have shown that CBRs and cannabinoids are not only responsible for psychoactive effects but also act as key mediators of crucial physiological processes, such as analgesia, immune regulation, appetite, nausea control, and mitigation of various pathologies. Many of these effects likely result from complex agonist/antagonist interactions involving CB1, CB2, and other GPCRs, yet the system’s complexity and interplay with other regulatory networks remain incompletely understood. Research on lesser-known phytocannabinoids—many of which demonstrate stronger biological activity than THC or CBD—shows particular promise for future therapeutic applications. While many fundamental mechanisms have already been explored, challenges remain in determining structure–function relationships, which is especially critical given that cannabinoid effects likely extend beyond CBRs. As a result, future studies will likely focus on their interaction with other GPCRs and related proteins.

Although many studies focus on the adverse effects of cannabis and its active compounds—particularly THC—it is important to distinguish between the physiological role of the endogenous cannabinoid system and the pharmacological impact of exogenous cannabinoids. The ECS is a tightly regulated signaling network involved in maintaining reproductive homeostasis. It modulates the HPG axis, gametogenesis, implantation, placental development, and early embryogenesis. The expression of cannabinoid receptors CBRs and endocannabinoid-metabolizing enzymes in reproductive tissues suggests a critical role in fertility and pregnancy maintenance.

However, cannabis exposure—especially chronic or high-dose use—has been associated with endocrine disruption, menstrual irregularities, ovulatory dysfunction, impaired spermatogenesis, and placental insufficiency. These observations do not merely reflect the toxic effects of THC but rather indicate that exogenous cannabinoids may dysregulate the finely tuned ECS, leading to downstream reproductive disturbances. In this context, THC acts as a functional probe that reveals the physiological relevance of ECS integrity. Therefore, the adverse reproductive outcomes observed in cannabis users should be interpreted not only as evidence of THC toxicity but also as indirect confirmation of the ECS’s physiological importance in reproductive regulation. This dual perspective enhances our understanding of how endocannabinoid tone and receptor signaling contribute to reproductive health and opens new avenues for therapeutic targeting of the ECS in infertility or pregnancy-related disorders.

Most existing research has focused on THC, with other cannabinoids remaining understudied. It is important to emphasize that cannabis-derived products commonly available on the market may contain complex cannabinoid profiles, sometimes with up to 60% of the extract composed of active cannabinoids. These formulations include not only CBD but also its acidic precursor CBDA (Cannabidiolic Acid), which exhibits different bioavailability and biological effects. Other co-occurring compounds include CBC, ∆8-THC, ∆9-THC, CBG, CBN, and their acidic forms [194,195,204,206].

Preliminary preclinical and animal studies involving species relevant to veterinary medicine, such as cats or ruminants, suggest that cannabinoids—particularly CBD—may provide health benefits in managing osteoarthritis, pruritus, epilepsy, and anxiety-related disorders. Their perceived natural origin and accessibility make them an attractive alternative to conventional pharmaceuticals. However, some suggested uses—such as for separation anxiety—still lack empirical confirmation. Moreover, despite increasing cannabinoid use in therapeutic contexts, comprehensive safety data remain limited, especially concerning reproductive health in both humans and animals. Of particular concern is the uncontrolled availability of cannabinoid products, leading to their unregulated use without medical supervision. Additionally, quality standards for these products vary significantly between countries, increasing the risk of exposure to unlabeled THC or contaminants in low-quality formulations. Although CBD does not produce intoxication like THC and is generally regarded as less toxic, it is psychoactive, and its long-term safety profile still requires further investigation. Observed side effects in animal studies have included gastrointestinal disturbances and alterations in liver enzyme activity, indicating the need for controlled and species-specific safety evaluations [233,234].

While CBD appears to be a promising therapeutic option, data regarding its effects—especially on reproductive physiology—are still scarce. Moreover, the physiological role of the ECS in reproduction itself remains incompletely understood and warrants further investigation. Most studies focus on rodent models, with even less information available for non-rodent species. Moreover, the physiological role of the ECS in reproduction itself remains incompletely understood and warrants further investigation. Moreover, many of the other bioactive compounds present in cannabis extracts have not yet been systematically evaluated. Available studies to date generally suggest potential negative effects of cannabinoids on reproductive health, underscoring the urgent need for detailed, species-specific investigations to draw reliable conclusions. Although this review includes studies on species relevant to veterinary medicine, it does not aim to provide a systematic overview of veterinary-specific cannabinoid research.

In conclusion, current evidence suggests that the ECS plays a crucial role in reproductive physiology. Exogenous cannabinoids—especially when used chronically or in uncontrolled doses—may disrupt this system and impair reproductive health. This underlines the need for cautious and evidence-based use of cannabinoids, as well as for further mechanistic and translational studies focusing on fertility, pregnancy, and endocrine regulation.

## Figures and Tables

**Table 1:** Location of cannabinoid receptors in the reproductive system and their known/potential effects on human and animal reproductive function.

Species	Receptor Subtype	Localization of Cannabinoid Receptors	Known/Potential Effects on
**Humans**	CB1	HPG axis *, Uterus, Testes, Spermatozoa, Mammary Gland	Hormonal regulation Mammary gland development/lactation Spermatogenesis disorders
	CB2	HPG axis *, Endometrium, Placenta	Embryo implantation Labor and neonatal development
**Non-human** **primates**	CB1	HPG axis *, Endometrium, Myometrium, Testes, Spermatozoa	Hormonal regulation Uterine contractility Sperm motility
	CB2	HPG axis *, Endometrium, Testes, Spermatozoa	Hormonal regulation Uterine contractility Spermatogenesis
**Cattle**	CB1	Corpus Luteum, Uterus	Modulation of luteal function Control of prostaglandin synthesis Regulation of uterine contractility
	CB2	Corpus Luteum, Uterine Immune Cells	Embryo implantation Immunomodulation and tolerance Anti-inflammatory effects
**Sheep**	CB1	Ovaries, Uterus	Steroid hormones synthesis Follicular maturation Control of prostaglandin release Endometrial receptivity
	CB2	Uterus, Uterine Immune Cells	Modulation of immune response Cytokine regulation Implantation support
**Pigs**	CB1	Uterus	Embryo implantation
	CB2	Placenta	Pregnancy maintenance
**Rodents**	CB1	HPG axis *, Uterus, Ovarian Follicles, Testes, Epididymis, Vas Deferens	Hormonal regulation Ovulation Gamete/embryo transport Embryo implantation Spermatogenesis disorders
	CB2	HPG axis *, Uterus, Endometrium, Ovarian Follicles, Placenta, Testes, Epididymis, Vas Deferens	
**Cats**	CB1	Ovaries, Ovarian Follicles	Ovulation Follicular development Potential luteal regulation
	CB2	Ovarian Follicles, Luteal Cells	Ovulation Potential luteal regulation

* HPG axis: hypothalamic–pituitary–gonadal axis. CB1 is more abundant in the central nervous system and reproductive tract, whereas CB2 is often found in immune-related cells, including those of the placenta and mammary gland [81,85,89,96]. Expression levels and functions vary depending on the phase of the estrous/menstrual cycle, gestation, and lactation stage [87]. Immunomodulatory roles of CB2 are especially relevant in the placenta and maternal–fetal interface [81,85,89,96].

**Table 2:** The role of the endocannabinoid system in reproductive functions in females.

Female Reproductive Function	ECS Component	Effects/Role
Ovulation	CB1, CB2, AEA, 2-AG	Modulation of GnRH, LH, and FSH secretion; involvement in follicular development and ovulatory peak
Ovarian function	CB1, CB2, AEA, 2-AG	Regulation of folliculogenesis, steroidogenesis, and oocyte maturation
Oviductal transport	CB1, AEA	CB1 modulates embryo transport; high AEA disrupts oviductal motility
Uterine receptivity and implantation	CB1, CB2, AEA	AEA gradient crucial for implantation; dysregulation linked to miscarriage
Pregnancy and placenta	CB1, CB2, AEA, 2-AG	Modulates trophoblast proliferation, vascularization, and immune tolerance; altered ECS linked to complications
Lactation	CB1, CB2, AEA	Inhibition of prolactin secretion via CB1; involvement in hypothalamic regulation
Menstrual/cycle regulation	CB1, CB2, AEA, 2-AG	Regulation of HPG axis, endometrial remodeling, and steroid secretion
Pathological states (e.g., endometriosis, PCOS)	CB1, CB2, AEA, 2-AG	Dysregulated ECS expression; potential target for modulation

**Table 3:** The role of the endocannabinoid system in reproductive functions in males.

Male Reproductive Function	ECS Component	Effects/Role
Spermatogenesis and testicular function	CB1, CB2, AEA, 2-AG	Regulation of Sertoli cell activity, spermatid maturation, and testosterone secretion
Epididymal maturation	CB1, CB2, AEA	Modulation of sperm motility and maturation; protective role against oxidative stress
Sperm capacitation and fertilization	CB1, CB2, AEA	High AEA impairs capacitation; CB1 controls acrosome reaction
Libido/sexual behavior	CB1, AEA	Inhibitory effects on hypothalamic-pituitary-gonadal axis; suppression of sexual drive
Hormonal regulation	CB1, AEA	Decreased LH, FSH, and testosterone secretion via hypothalamic-pituitary modulation
Seminal plasma ECS content	AEA, 2-AG	Detected in semen; possible role in sperm activation and immune tolerance
Pathological states (e.g., infertility, varicocele)	CB1, CB2, AEA, 2-AG	ECS imbalance linked to poor sperm quality and reproductive disorders

## Data Availability

No new data were created or analyzed in this study. Data sharing is not applicable to this article.

## Abbreviations

The following abbreviations are used in this manuscript:

2-AG	2-Arachidonoylglycerol
AEA	Anandamide
Act1	NF-κB Activating Protein 1
AI	Aromatase Inhibitor
AIDS	Acquired Immunodeficiency Syndrome
AKT	Protein Kinase B
BPA	Bisphenol-A
cAMP	Cyclic Adenosine Monophosphate
CBC	Cannabichromene
CBD	Cannabidiol
CBDA	Cannabidiolic Acid
CBG	Cannabigerol
CBN	Cannabinol
CBR	Cannabinoid Receptor
CB1	Cannabinoid Receptor type 1
CB2	Cannabinoid Receptor type 2
CDK1	Cyclin-Dependent Kinase 1
CL	Corpus Luteum
CNS	Central Nervous System
COX-1	Cyclooxygenase type 1
COX-2	Cyclooxygenase type 2
DINP	Diisononyl phthalate
E2	Estradiol
ECS	Endocannabinoid System
EDC	Endocrine-disrupting chemical
EGF	Epidermal Growth Factor
EGFR	Epidermal Growth Factor Receptor
ER-	Estrogen Receptor Negative
ER+	Estrogen Receptor Positive
ERα	Estrogen Receptor Alpha
ERβ	Estrogen Receptor Beta
ERK	Extracellular Signal-Regulated Kinase
EVSA-T	Estrogen-Responsive Mammary Tumor Cell Line
FAAH	Fatty Acid Amide Hydrolase
FDA	Food and Drug Administration
FSH	Follicle-Stimulating Hormone
GCNIS	Germ cell neoplasia in situ
GnRH	Gonadotropin-Releasing Hormone
GPCR	G Protein-Coupled Receptor
HER2	Human Epidermal Growth Factor Receptor 2
HER2+	Human Epidermal Growth Factor Receptor 2 Positive
HIV	Human Immunodeficiency Virus
HPG axis	Hypothalamic–Pituitary–Gonadal Axis
Id-1	Inhibitor of Differentiation Protein 1
LH	Luteinizing Hormone
MAGL	Monoacylglycerol Lipase
MAPK	Mitogen-Activated Protein Kinase
MMTV-neu	Mouse Mammary Tumor Virus-neu
mRNA	Messenger Ribonucleic Acid
mTOR	Mechanistic Target of Rapamycin
NAM	Negative Allosteric Modulator
NGF	Nerve Growth Factor
NPS	New Psychoactive Substances
NSAID	Non-Steroidal Anti-Inflammatory Drug
OSAS	Obstructive Sleep Apnea Syndrome
P4	Progesterone
PCOS	Polycystic Ovary Syndrome
PGE	Prostaglandin E
PGE_1_	Prostaglandin E1
PGE_2_	Prostaglandin E2
PI3K	Phosphatidylinositol-3-kinase
PKC	Protein Kinase C
PPAR	Peroxisome Proliferator-Activated Receptor
PR	Progesterone Receptor
PR-	Progesterone Receptor Negative
PR+	Progesterone Receptor Positive
PRL	Prolactin
PTSD	Post-Traumatic Stress Disorder
ROS	Reactive Oxygen Species
SC	Synthetic Cannabinoid
SERM	Selective Estrogen Receptor Modulator
T	Testosterone
TDS	Testicular dysgenesis syndrome
THC	∆9-Tetrahydrocannabinol
THCV	Tetrahydrocannabivarin
TNBC	Triple-Negative Breast Cancer
TRPV1	Transient Receptor Potential Vanilloid 1
VEGF	Vascular Endothelial Growth Factor
